# Tsunami: Ocean dynamo generator

**DOI:** 10.1038/srep03596

**Published:** 2014-01-08

**Authors:** Hiroko Sugioka, Yozo Hamano, Kiyoshi Baba, Takafumi Kasaya, Noriko Tada, Daisuke Suetsugu

**Affiliations:** 1 Japan Agency for Marine-Earth Science and Technology, Yokosuka, Japan; 2 Earthquake Research Institute, University of Tokyo, Tokyo, Japan

## Abstract

Secondary magnetic fields are induced by the flow of electrically conducting seawater through the Earth's primary magnetic field (‘ocean dynamo effect’), and hence it has long been speculated that tsunami flows should produce measurable magnetic field perturbations, although the signal-to-noise ratio would be small because of the influence of the solar magnetic fields. Here, we report on the detection of deep-seafloor electromagnetic perturbations of 10-micron-order induced by a tsunami, which propagated through a seafloor electromagnetometer array network. The observed data extracted tsunami characteristics, including the direction and velocity of propagation as well as sea-level change, first to verify the induction theory. Presently, offshore observation systems for the early forecasting of tsunami are based on the sea-level measurement by seafloor pressure gauges. In terms of tsunami forecasting accuracy, the integration of vectored electromagnetic measurements into existing scalar observation systems would represent a substantial improvement in the performance of tsunami early-warning systems.

The induction of secondary magnetic fields by the flow of electrically conducting seawater through the Earth's ambient magnetic field, by a physical process called the ‘ocean dynamo effect’, has been known since the phenomenon was first postulated by Faraday in 1832^1^. There are several reports on the phenomena of ocean water transport monitored by submarine cables^2^,^3^ and ocean tidal signal extracted from satellites data^4^,^5^. In principle, tsunami propagation should also generate secondary magnetic fields, although the amplitudes would be very low^6^,^7^. The feasibility of using electromagnetic (EM) sensors to detect tsunamis has been discussed in the context of in situ seafloor measurements of the electric field using submarine cables^6^,^8^ and remote sea surface tracking of the magnetic field by satellites^9^; however, it has been concluded that magnetospheric disturbances might limit electromagnetic tsunami monitoring to periods of low solar activity, as the expected tsunami signals are episodic and weak and their frequencies overlap with those of other external magnetic sources^6^.

Many studies on motional induction in oceans^6^,^7^,^9^,^10^,^11^,^12^ have been conducted since the first comprehensive theoretical study by Longuet-Higgins et al. was published in 1954^13^. In most formulations, an electrostatic approximation that considers the weakness of the self-induction effect is used; however, the approximations are not suitable for tsunami flows, as tsunami phase velocities of ~200 m/s are much greater than electromagnetic diffusion speeds in deep oceans of ~10 m/s^7^. For tsunamis in relatively high frequencies of ~10 min propagating thorough deep oceans such of ~5000 m, it is inapplicable to assume that water depth is much larger than a skin depth of ~6500 m as the previous study applyed^9^. Electromagnetic induction by motional sources near the seafloor has been theoretically examined by taking into account the self-induction term, and the theory is applicable to the analysis of tsunami-induced EM signals recorded at seafloor observatories. We briefly present the theoretical basis for tsunami-induced EM signals (see Methods), in which we examine the theoretical response function of the vertical magnetic field perturbation *b_z_*to sea level change *η* (see Equation (5) below). Figure 1a and b shows the amplitude and phase of the right hand side (RHS) of the conversion function, expressed by 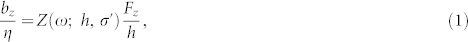
where *Z* is a function of the period *ω*, water depth *h*, and electrical conductivity (in this case, the conductivity of the layer below the seafloor is *σ*′ = 0), and *F_z_* is the static magnetic field. Equation (1) describes the proper scaling and phase lag between *b_z_*and *η*. Here, we use the vertical geomagnetic field model IGRF-11^14^ as *F_z_*, and we use the global bathymetry model ETOPO1^15^ as *h*; the electrical conductivity of the layer below the ocean is set to zero. Note that the phase lag represented by *Z*(*ω*; *h*, *σ*′) is always negative, indicating that the magnetic response precedes sea-level signals.

The recent development of high-precision electromagnetometers by our group has enabled seafloor detection of EM fields associated with the passage of tsunamis^16^. Our data were obtained during the Mw 8.8 earthquake event that occurred off the coast of Chile (35.846°S, 72.719°W) at 0634 UT on 27 February 2010, which triggered a tsunami that propagated across the Pacific Ocean. We detected the tsunami-induced perturbations of the seafloor EM field and water depth using electromagnetometers and a pressure gauge, respectively, installed in our long-term seafloor geophysical observational network in the French Polynesia region, which was operated from February 2009 to December 2010^17^. Here, we verify the theoretical considerations presented above with the first simultaneous measurements of tsunami-induced sea level changes and seafloor EM field perturbations, while the induction magnetic field recorded on Easter Island observatory has been previously reported^18^. Figure 1 shows the locations of the earthquake epicentre and the observational network. The network, located 7000 km west of the earthquake epicentre, consisted of nine pairs of ocean bottom electromagnetometers (OBEM) and ocean bottom broadband seismometers at Sites 1–9, and a differential pressure gauge (DPG) at Site 8^19^, dispersed over distances of ~400 km in east–west and north–south directions. The earthquake-induced tsunami arrived at the network region ~10 hours after the earthquake event.

During passage of the tsunami waves, the DPG at Site 8 detected a bottom pressure change of 1200 Pa, which corresponds to a sea level change of ~12 cm (assuming a linear pressure–water height response), and the nine OBEMs recorded vertical perturbations of the electromagnetic field; the amplitudes of the vertical magnetic and the horizontal electric field perturbations associated with the first tsunami wave were ~0.5 nT and ~0.15 μV/m, respectively, values which are ~50 and ~2 times greater, respectively, than the resolution limits of the instruments (0.01 nT and 0.07 μV/m, respectively). Figure 2 shows that the vertical magnetic perturbations and corresponding sea level changes associated with the tsunami (recorded at Site 8) lasted for several hours after the first motion, and that the coherence of the signal perturbations was strong throughout the duration of the event. Interestingly, the magnitude of the vertical magnetic perturbation induced by the tsunami *b_z_* relative to the static geomagnetic field *F_z_* (*b_z_*/*F_z_* = 0.5/19,500 ≈ 0.0000256) was almost coincident with that of the sea level change due to the tsunami *η* relative to sea depth *h* (*η*/*h* = 0.12/4800 ≈ 0.0000250), which verify the theoretically expected relationship when *Z*(*ω*; *h*, *σ*′) ≈ 1 (Equation (1)), a condition that corresponds to values of *h* = 4800 m, a measured electrical conductivity of *σ*′ = 10^−2^ S/m^20^, and a tsunami period of 2400 s (see Equation (6) and Figure 6 of the Methods). In the case of *Z*(*ω*; *h*, *σ*′) = 1, we obtain a simple relationship among dimensionless quantities (see Equation (5) of the Methods for details): 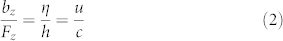
where *u* is the particle velocity and *c* is the phase velocity of the tsunami.

The EM signals at all nine sites of the network were evident in the three components of the magnetic field (*b_x_, b_y_*, *b_z_*) and the two horizontal components of the electric field (*e_x_*, *e_y_*), and the variations of the dispersed tsunami signals lasted for more than several hours after the passage of the first tsunami wave. The propagation characteristics of the tsunami wave over the network could be accurately restored from the EM sensor array, based on the coherence of the EM field variations among the nine stations. Figure 3a shows the inferred wave fronts of the tsunami at intervals of 10 min, based on arrival times at the stations, indicating tsunami propagation towards approximately N44°W at ~210 m/s. It also shows the wavenumber vectors estimated from the two horizontal magnetic and electric field components as shown in Figure 3b, based on the theoretical relationships presented in Equation (4) of the Methods, indicating tsunami propagation towards N43°W at an average speed of 239 m/s. Although these characteristics of the tsunami propagation are independently estimated, they are consistent with each other, suggesting that the tsunami propagated through the network region at an average water depth of 4000–4800 m with a speed *c* given by the long-wave approximation *c* = (*gh*)^1/2^, where *g* is the gravitational acceleration. We then calculated the particle velocity of seawater as ~5 cm/s using Equation (4) of the Methods.

Recently, it has been reported that a reversed prolonged initial phase precedes the main phase arrival of tsunami waveforms traversing deep oceans, represented by a very small (though visible) signal in tidal pressure data. It is thought that the reversed phase is caused by the dispersion of the tsunami, especially related to the effects of elastic loading of the substrate by the tsunami^21^,^22^. We identified the reversed initial phases in the magnetic data for the 2010 Chilean earthquake tsunami, as indicated by the black arrows in Figure 4. The reversal is more pronounced in the vertical magnetic field data than in the pressure signal recorded by the DPG at Site 8, as the response of the vertical magnetic field is almost flat at periods longer than 1000 s on account of a low-pass filter effect, as shown by the theoretical response functions (see Equation (6) and Figure 5 of the Methods). Therefore, compared with the bottom pressure signal, the EM field data may be of greater utility for identifying the initial reversal phase preceding the main phase of a tsunami.

As shown above for the 2010 Chilean earthquake tsunami, our recent development of high-precision sensors for EM field detection enabled us to detect tsunami-induced perturbations of the deep seafloor EM field, and to verify the theoretical basis for the perturbations, despite the fact that the sensors were originally developed for detection of the Earth's EM field. The resolution limits of our seafloor electromagnetometer of 0.01 nT are sufficiently small to resolve tsunamis with cm-order heights corresponding to *b_z_*/*η* > 1, and this condition is satisfied in wide areas except the equator line in Figure 1a (as derived from theoretical considerations). Theoretical relationships also demonstrate that observations of the EM field with two electric and three magnetic field components, even at a single station, can extract information about variations in sea level associated with tsunami flows, the propagation directions of tsunami waves, and the phase velocity of tsunami propagation and its frequency dependency (the dispersion relation). Presently, offshore sea-level measurements based on seafloor pressure gauges provide information for the early forecasting of tsunamis prior to their arrival on coastlines, though tsunami detection using the dynamo effect in ionosphere has been suggested since the 2004 Sumatra earthquake tsunami^23^,^24^. Because the EM signals induced by tsunami flows record information on the propagation vector of the tsunami, as well as the particle motions of the seawater, the EM signals can supplement data on sea-level changes inferred from bottom pressure gauges; EM measurements may become indispensable for monitoring tsunami propagation in open oceans, and utilized for early warning of tsunami arrival at on-shore locations.

## Methods

Herein, we present a theory of tsunami-induced electromagnetic fields on the deep-sea floor. We consider tsunami motions in an unbounded ocean layer of constant depth *h* underlain by a semi-infinite layer, with magnetic diffusion coefficients of *K* = (*μσ*)^−1^ and *K*′ = (*μσ*′)^−1^, respectively, where *μ* is the magnetic permeability, and *σ* (~4 S/m) and *σ*′ (~0.001–1 S/m)^25^ are the electrical conductivities of the respective layers. The sea surface is in contact with insulating air. The equation of magnetic induction which considers the self-induction effect is ∂**b**/∂*t* = ∇ × (**u** × **F**) − ∇ × (*K*∇ × **b**), where **b** and **F** are the motionally induced and the Earth's ambient magnetic fields, respectively, which obey ∂**F**/∂*t* = 0, ∇ × **F** = 0, ∇^2^**F** = 0 and |**b**| ≪ |**F**|, and **u** is the flow velocity. While the flow is predominantly along geopotential surfaces, we assume that |**u***_H_*| ≫ **u***_z_* (long-wave approximation). We then solve the induction equation by assuming the solution as a plane wave of 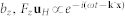 in the wavenumber–frequency (**k** − *ω*) domain, where **x** is the horizontal position vector. Combination of the above equations yields a solution for the vertical magnetic field which, evaluated at the seafloor, is 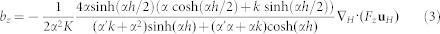
where *α*^2^ = *k*^2^ − *iω*/*K* and *α*′^2^ = *k*^2^ − *iω*/*K*′. Using the Maxwell equation ∇·**b** = 0, the horizontal component of the magnetic field is evaluated as *b_H_* = −(*ik*)^−1^∂*b_z_*/∂*z*.

Furthermore, using the Maxwell equation ∂**b**/∂*t* = −∇ × **e**, where **e** is the induced electric field, the two horizontal components of the electric field are obtained from the expression for the induced magnetic fields. When the propagating direction of tsunamis is given as **k** = (*k_x_*, *k_y_*), we have 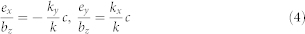
where *c* is the phase velocity obeying *c* = (*g*tanh(*kh*)/*k*)^1/2^, where *g* is the gravitational acceleration.

Under the long-wave approximation, the continuity equation for the flow allows the expression ∇·(*F_z_***u***_H_*) = *iωηF_z_*/*h*, where *η* is the sea level change. Therefore, we obtain the magnetic perturbation corresponding to the sea level change at the seafloor by introducing conversion functions of in terms of *Z* and *H*: 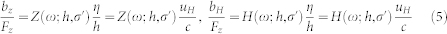
where 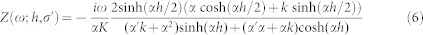

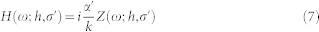


**Figure 5:**
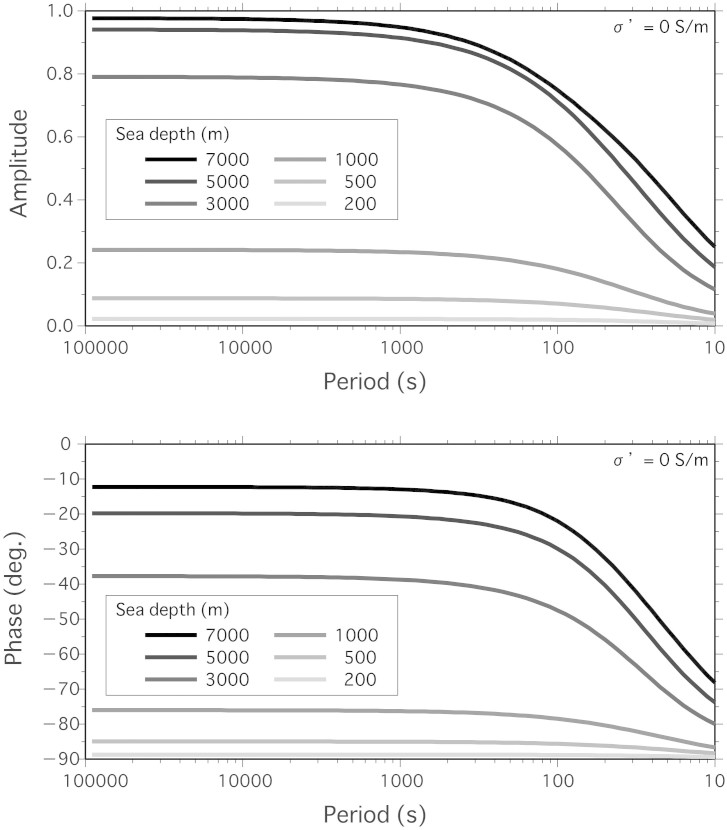
Response function of the magnetic field for different sea depths. Showing amplitude (top) and phases (bottom) of the response function of the vertical magnetic field *Z*(*ω*; *h*, *σ*′) as a function of the period ( = 2*π*/*ω*) (as shown in Equation (5) and (6), respectively of the Methods) for the depths *h* = 7000, 5000, 3000, 1000, 500, and 200 m, and for *σ*′ = 0.

**Figure 6:**
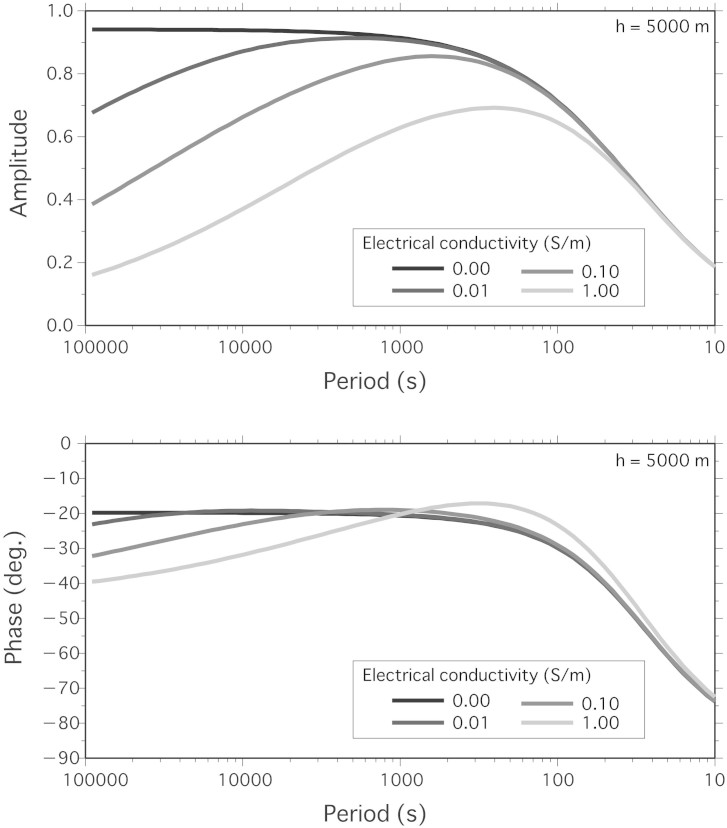
Response function of the magnetic field for variable electrical conductivities of the layer underlying the ocean. See the Figure 5 legend for details; the electrical conductivities are *σ*′ = 0.00, 0.01, 0.10, and 1.00 S/m, and *h* = 5000 m.

These magnetic response functions are shown in Figures 5 and 6.

## Author Contributions

H.S. observations, data analysis and theory; Y.H. theory and data analysis; K.B. observations; T.K. observations; N.T. observations; D.S. project planning.

## Figures and Tables

**Figure 1:**
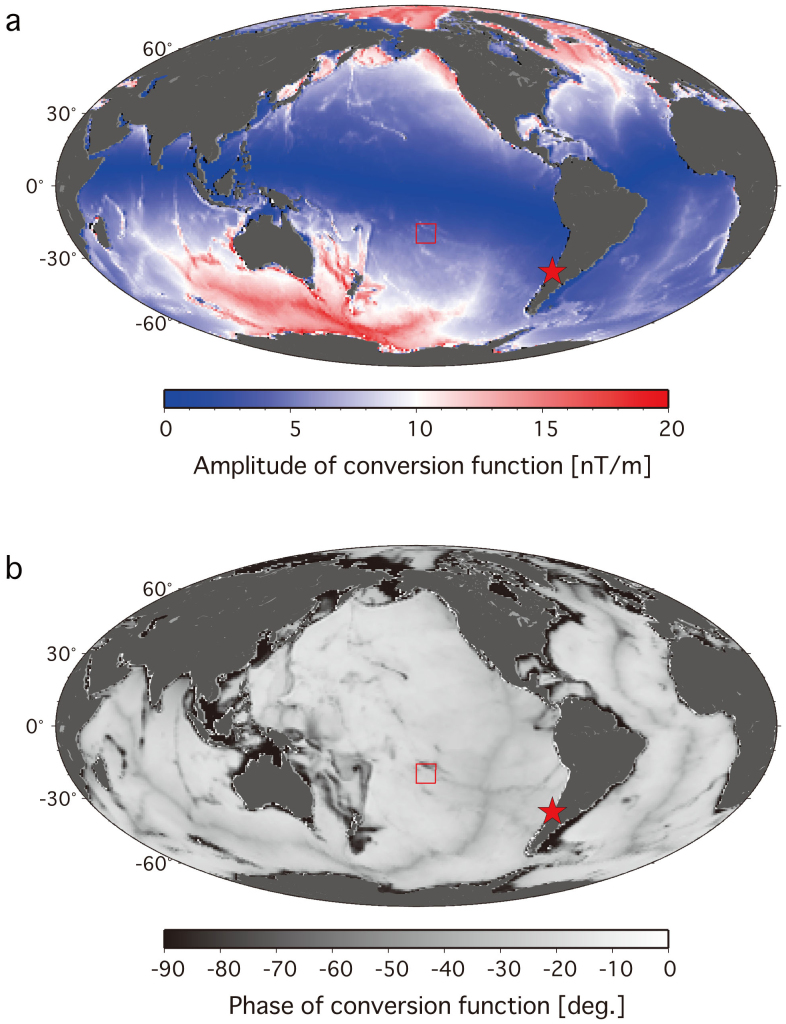
Conversion function of the magnetic field perturbations associated with sea-level changes. The function is theoretically estimated using the vertical geomagnetic field model IGRF-11^14^ and the global bathymetry model ETOPO1^15^. The amplitude and the phase of the function are shown in (a) and (b), respectively. The red star shows the location of the 2010 Chilean earthquake (Mw 8.8) epicentre. The red rectangle shows the location of our seafloor array network^17^. The figure was generated using Generic Mapping Tool (http://gmt.soest.hawaii.edu/).

**Figure 2:**
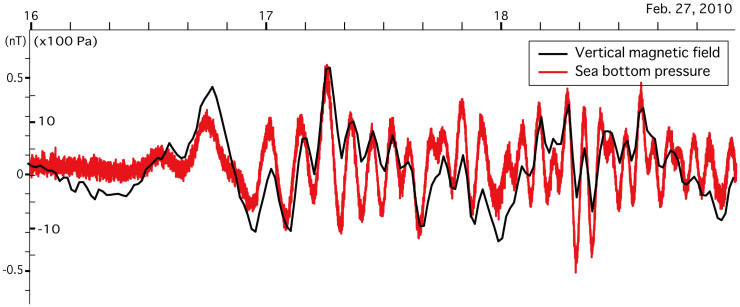
Observed magnetic field perturbations and sea level changes associated with the 2010 Chilean earthquake tsunami. Observed vertical magnetic field perturbations (black) and sea bottom pressures (red) induced by the tsunami at a depth of 4800 m at Site 8 of the seafloor array network. The tsunami arrived at the network region at 1600–1700 on 27 February. The sea-bottom pressure recorded by the differential pressure gauge (DPG) was corrected for the frequency response^19^ and the in situ amplitude response of predicted ocean tides^26^. The waveforms were high-pass filtered at 0.0002 Hz. The magnetic field perturbation and the seafloor pressure change associated with the tsunami were ~0.5 nT and ~1200 Pa, respectively.

**Figure 3:**
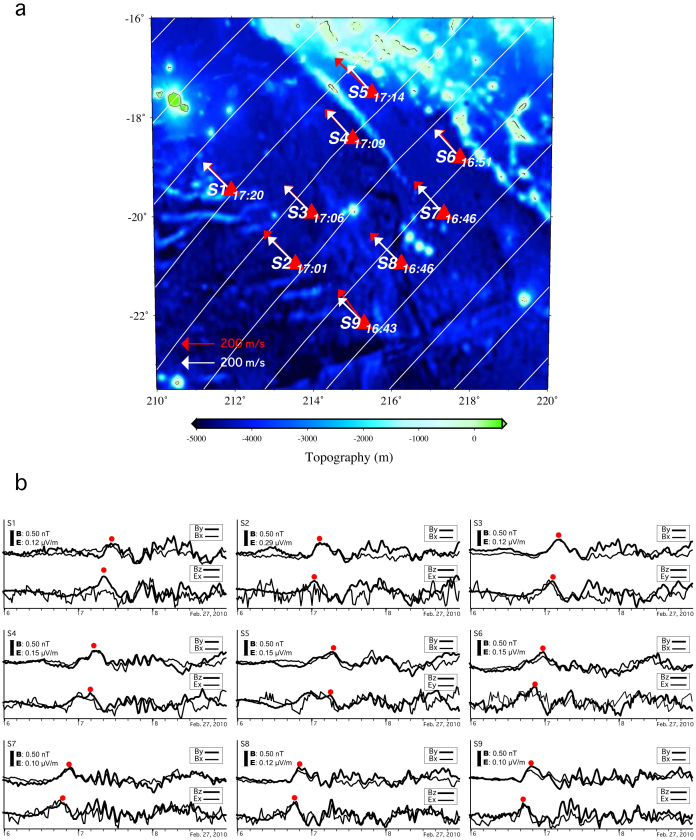
Tsunami wave fronts associated with the 2010 Chilean earthquake inferred from arrival times at the seafloor electromagnetometer array network. (a) Arrival times of the tsunami (earthquake origin time: 0634 on 27 February) at the nine-station array in our seafloor network in the French Polynesia region^17^. White lines and white arrows show the estimated positions of the tsunami wave front at 10-min intervals and the tsunami propagation vectors, respectively, based on the arrival time at all sites. Red arrows show the propagation vectors inferred from the electromagnetic data shown in (b), which were towards N43°W at an average phase speed of 239 m/s. (b) Two-pair waveforms of the two horizontal magnetic components, and the one horizontal electric component and the vertical magnetic component for all stations. These data were used to estimate the propagation vector of the tsunami, based on the theoretical relationship given by Equation (4) of the Methods.

**Figure 4:**
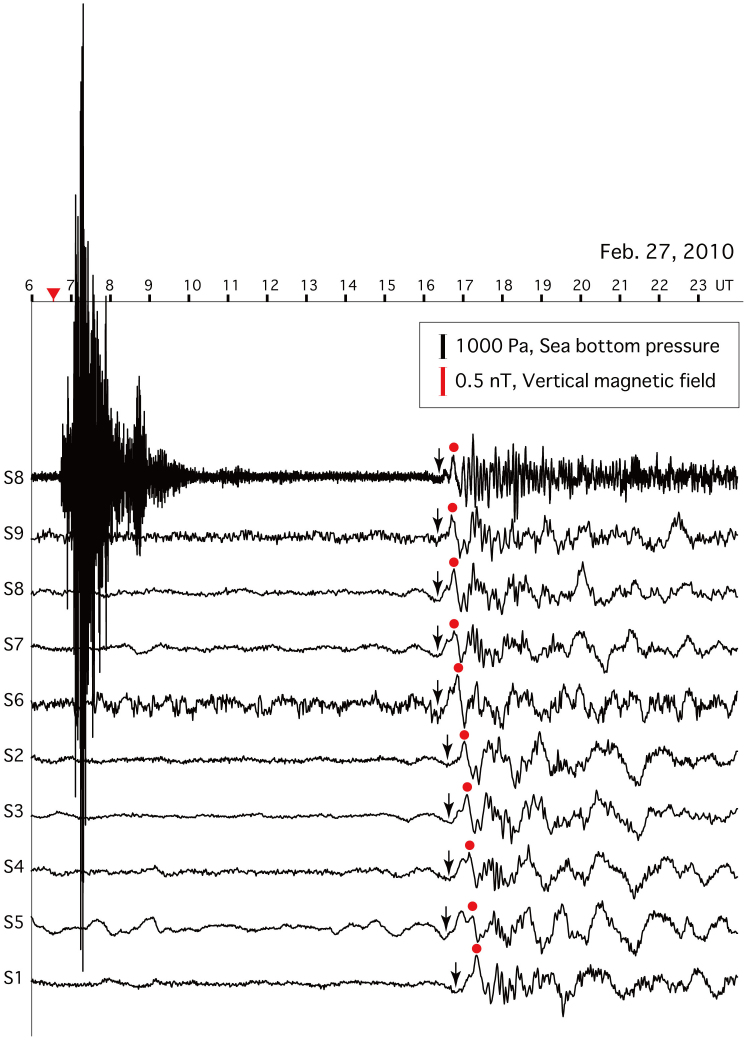
Tsunami-induced perturbations of the seafloor electromagnetic field and pressure recorded at the seafloor array network. Pressure perturbations were recorded using a differential pressure gauge (DPG) at Site 8 (top), and the vertical magnetic field perturbations were recorded by electromagnetometers at all nine stations. The tsunami was associated with the 2010 Chilean earthquake, which occurred at 0634 on 27 February (red triangle). The first arrival signal recorded by the DPG was caused by the seismic wave. The tsunami arrived at the network ~10 hours after the event. All waveforms were high-pass filtered at 0.0002 Hz. The sea-bottom pressure recorded by the differential pressure gauge (DPG) was corrected for the frequency response^19^ and the in situ amplitude response of predicted ocean tides^26^. Black and red dots show the peak of the main phase of the tsunami. Black arrows indicate the reverse peak of the initial phase preceding the positive peak of the main phase of the tsunami.
